# Dr. William Pepper

**Published:** 1898-09

**Authors:** 


Dr. William Pepper, of Philadelphia, died suddenly of angina pectoris on July 28, 1898, at Mrs. Hearst's country residence near Pleasanton, California, whither he went to recuperate after an unusually laborious winter. His father was a physician and he was born at Philadelphia, August 21, 1843, hence was near 55 years of age. He took his degree in arts at the University of Pennsylvania in 1862 and his degree in medicine in 1864. He became a teacher in his Alma Mater in 1868 and was elected provost thereof in 1881. At the time of his death he held the chair of theory and practice of medicine, but he resigned as provost in 1894, at which time he presented the University with $50,000. He was a member of several medical societies—local, state, national and international,—was president of the first Pan-American Medical Congress, 1893, as well as president of the third, which is to convene in Venezuela two years hence. He was a medical writer of well-known capacity, a teacher of renown, an executive officer of great skill and judgment and a physician whose services were in large demand.

